# Geographical Isolation, Buried Depth, and Physicochemical Traits Drive the Variation of Species Diversity and Prokaryotic Community in Three Typical Hypersaline Environments

**DOI:** 10.3390/microorganisms8010120

**Published:** 2020-01-16

**Authors:** Shaoxing Chen, Yao Xu, Libby Helfant

**Affiliations:** 1 College of Life Sciences, Anhui Normal University, No.1 Beijing East Road, Wuhu 241000, China; 2 College of Life Sciences, Honghe University, No.1 Xuefu Road, Mengzi 661100, China; 3 Department of Molecular Biology and Biochemistry, Rutgers, The State University of New Jersey, Piscataway, NJ 08854, USA; ljh114@scarletmail.rutgers.edu

**Keywords:** hypersaline environment, microbial community, species diversity, clone library, high-throughput sequencing, 16S rRNA gene

## Abstract

The prokaryotic community composition, species diversity and the distribution patterns at various taxonomic levels in a salt lake (Chaka salt lake), solar salterns (Taipei saltworks and Dongfang saltworks), and salt mines (Yuanyongjing salt mine, Xiangyan salt mine, and Dinyuan salt mine) were investigated using clone library or Illumina MiSeq sequencing. The clone library approach revealed that the salt lake harbors low species diversity (H’ = 0.98) as compared to the solar saltern (H’ = 4.36) and salt mine (H’ = 3.57). The dominant group in the salt lake is a species from the genus *Haloparvum* which constitutes about 85% of the total sequences analyzed. The species diversities in salt salterns and salt mines are richer than in the salt lake, and the dominant group is less significant in terms of total percentage. High-throughput sequencing showed that geographical isolation greatly impacted on the microbial community (phyla level) and species diversity (operational taxonomic units (OTUs) level) of salt mines. Species of the genus *Natronomonas* are found in all three types of environments investigated. In addition, the microbial community and species diversity of different stratums of the salt mine are very similar. Furthermore, species of the genus *Halorubrum* flourish in the newest stratum of salt mine and have become the dominant group. This study provides some new knowledge on the species diversity and prokaryotic community composition of three typical hypersaline environments.

## 1. Introduction

Hypersaline environments are distributed all around the world [1] and are influenced by different environmental conditions, such as low temperatures as in the Deep Lake in Antarctica or relatively high temperatures as in the Dead Sea [2]. Numerous studies have revealed microbial diversity of different types of hypersaline environments, including salt lakes [3,4,5], solar salterns [6,7], and salt mines [8,9,10] using culture-dependent or culture-independent approaches.

Salt lakes have the highest fluidity, followed by solar salterns and salt mines. Microbial diversity in salt lakes fluctuates drastically during different seasons [11]. Ion composition and salt concentration have been proposed to be the main factors driving the microbial community structure in different salt lakes of the Tibetan Plateau [12]. Seasonal dynamics of microbial communities have also been reported in solar salterns [2]. Differing from the salt lake and solar salterns, the physicochemical traits of salt mines varied among stratums and buried depths with perennially stable ions compositions and salt concentrations. Variation of salt concentrations and ion compositions in different seasons will significantly change the composition of microbial communities. A clone library strategy based on 16S rRNA gene revealed that the prokaryotic diversity of two salt mines in Yunnan Province, China (about 220 km apart), namely Yipinglang Salt Mine and Qiaohou Salt Mine, are significantly different from each other [10]. Furthermore, a culture-dependent approach adopted to isolate halophilic microorganisms from ancient halite samples with various buried depths [13] resulted in isolation of very few halophiles. The culture-independent approach was considered a more powerful strategy in unveiling the microbial communities in different stratums of salt mines.

The microbial communities in salt lakes, solar salterns, and salt mines have been explored widely using various approaches, though parallel comparison among these different hypersaline environments is rare. In addition, our knowledge of the structure of microbial communities in different stratums of salt halite is also limited. Hence, this study aims to perform a comprehensive comparison of salt lakes, solar salterns, and salt mines with varying buried depths at a wide range of different locations throughout Anhui province. This is done primarily through utilizing the clone library strategy and high-throughput sequencing of the 16S rRNA gene. We found that the species diversity of solar salterns is slightly greater than that of salt mines, while species diversity of salt lakes is the lowest. In addition, samples taken closer to each other have a more similar microbial community structure than samples taken farther away from each other. The occurrence of haloarchaea at the genus level is significantly different at different buried depths, which may attribute to the continual speciation and extinction among haloarchaeal species in extremely hypersaline environments.

## 2. Materials and Methods

### 2.1. Sample Collection

**Figure 1:**
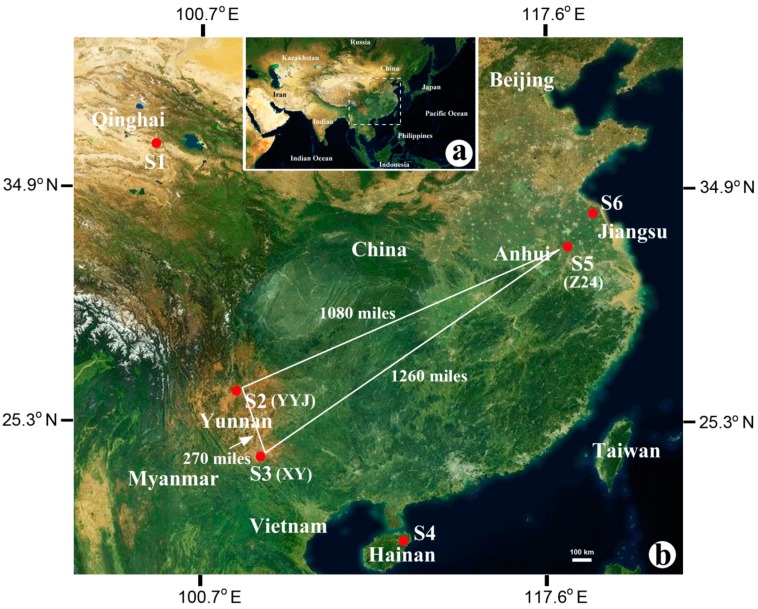
Location of the sampling sites. (**a**) Large scale map including sampling sites. (**b**) Close-up of the sampling area showing six different sampling sites from S1 to S6. These six sampling sites were grouped into three types: salt lake, salt mine, and solar saltern. S1: salt lake; S2, S3, and S5: salt mine; S4 and S6: solar saltern. The distances between each site of S2, S3, and S5 are shown.

The salt lake sample (salt brine; fluid) was collected from Chaka Salt Lake (CK) (3059 m above sea level) in Qinghai Province, China (Table 1 and Figure 1). The salt mine samples (rock salt) were collected from Yuanyongjing Salt Mine (YYJ) and Xiangyan Salt Mine (XY) in Yunnan (China) and Dinyuan Salt Mine (Z24-1, Z24-6, and Z24-8) in Anhui (China) (Table 1 and Figure 1). The solar saltern samples (saline soil) were collected from Taipei saltworks (LYG) in Jiangsu (China) and Dongfang saltworks (HND) in Hainan (China) (Table 1 and Figure 1). Salt crystal samples from different buried depths (Z24-1, Z24-6, and Z24-8) were collected from the Dinyuan Salt Mine in Anhui (China) (Table 1 and Figure 1).

### 2.2. Chemical Analyses of Salt Mines

Analysis of the chemical composition of samples taken from the salt mine was based on standardized methods as performed by a commercial analytical laboratory (Sci-tech innovation, Qingdao, China). The concentration of hydronium ion (pH value) was determined by a pH meter (Mettler Toledo, MP225, Shanghai, China). Sulfate (SO_4_^2^^−^) and chloride (Cl^−^) concentrations were measured by ion chromatography on ICS-1500 (Dionex, Sunnyvale, CA, USA). The concentrations of elements Mg, Fe, Mn, Ca, Na, and K were determined by inductively coupled plasma atomic emission spectrometry (ICP-AES) using OPTIMA 5300 DV spectrometer (Perkin Elmer, Norwalk, USA). The total nitrogen content (TN) as bound nitrogen (including free ammonia, ammonium, nitrite, nitrate, and organic nitrogen but excluding dissolved nitrogen gas) was assessed by combustion followed by oxidation to nitric dioxide and subsequent chemiluminescence detection. Total organic carbon (TOC) was determined by sample acidification followed by combustion and IR detection of CO_2_ released. Both parameters (TN and TOC) were measured according to EN 12260 and EN 1484, respectively, using the multi N/C 2100 S Analyzer (Analytik Jena, Jena, Germany).

### 2.3. Total Environmental DNA Extraction

Total environmental DNA extraction was conducted on 100 g of the sample from the salt mines and solar salterns dissolved in 500 mL of sterilized 10% (*w*/*v*) NaCl solution as well as 500 mL of the brine collected from the salt lake [14]. For the collection of microorganisms, saline water samples were filtered through a 0.22 µm pore size Nylon syringe filter (Millipore, MA, USA). Filters were collected after they were filled to capacity and blocked. Five blocked filters for each sample were then cut into pieces and mixed with extraction buffer (100 mM Tris-HCl, 100 mM EDTA pH 8.0) with lysozyme (1 mg mL^−1^) and RNase A (10 μg mL^−1^), then incubated at 37 °C for 2 h. Then, proteinase K (stock solution: 150 mg mL^−1^; final concentration: 10 mg mL^−1^) and 10% sodium dodecyl sulfate (final concentration: 1%) were added and the mixture was incubated at 37 °C for 2 h after suspension. Next, an equal volume of CTAB solution (4% CTAB, 0.5 M NaCl) was added to the mixture, and then incubated at 65 °C for another 2 h. DNA extraction and purification were performed using phenol-chloroform-isoamyl alcohol (25:24:1) followed by ethanol precipitation and resuspension in sterile Milli-Q water. Total DNA samples were stored at −80 °C for further use after measuring the concentration and purity with NanoDrop-2000 (Thermo, MA, USA). DNA concentration and purity were determined by monitoring the optical density (OD) at 260 and the ratio of OD_260_/OD_280_ [15]. The concentration of the total environmental DNAs isolated from different samples was adjusted to about 50 μg mL^−1^ (1 OD_260_ = 50 μg mL^−1^) with distilled water for PCR and high-throughput sequencing. The purities of different samples of total environmental DNAs calculated from the ratio of OD_260_/OD_280_ were between 1.89 and 1.96.

### 2.4. Clone Library Construction and DNA Sequencing

PCR amplification of the 16S rRNA gene was performed using the total environmental DNA (≈100 ng) as a template, and F8 (5′-TTGATCCTGCCGGAGGCCATTG-3′) and R1462 (5′-ATCCAGCCGCAGATTCCCCTAC-3′) [16] as the forward and reverse primers (Table 2). The PCR amplification was performed on ABI 3730 Thermal Cycler under the following PCR procedure: 94 °C for 2 min, followed by 30 cycles of 94 °C for 45 s, 53 °C for 1 min, and 72 °C for 1.5 min, with a final extension at 72 °C for 10 min. The PCR products were inserted into pMD18T vector (TaKaRa, Japan) for DNA sequencing after PCR amplification and DNA purification. Determination of the taxonomic position of each DNA sequence was performed using a similarity search against the public database BLAST (https://blast.ncbi.nlm.nih.gov/Blast.cgi).

### 2.5. High-Throughput Sequencing

Primer pair F338 and R806 (Table 2) were used to generate the 16S rRNA gene amplicons [17]. High-throughput sequencing of the V3 and V4 regions of the 16S rRNA gene was conducted using Illumina HiSeq 2500 (BioTeke, Beijing, China). The original reads were assembled with the FLASH (Fast Length Adjustment of SHort reads) (version 1.2.11), filtrated with the Trimmomatic (version 0.33) [18,19]. Then, high-quality sequence data for further analysis were obtained by removing the chimeras with software UCHIME (version 8.1) [20].

### 2.6. Operational Taxonomic Unit (OTU) Assignment

Sequences with a similarity value greater than 97% were assigned to one operational taxonomic unit (OTU). OTUs with a richness of less than 0.005% were removed [21]. Taxonomic assignments were performed using RDP Classifier platform (http://sourceforge.net/projects/rdpclassifier/) (confidence coefficient, 0.8) based on the Silva database, Release132 (http://www.arb-silva.de) [22].

### 2.7. Species Diversity Evaluation

Rarefaction curves were generated using the software MOTHUR version v.1.30 (http://www.mothur.org/). The community richness was evaluated by Chao1 and ACE (abundance-based coverage estimation). The species diversity was analyzed using the Shannon index and Simpson index. All these estimations of alpha diversity metrics (Chao1, ACE, Shannon Index, and Simpson Index) were performed using MOTHUR. Beta diversity was evaluated by the PCA (principal components analysis), UPGMA (un-weighted pair-group method with arithmetic means), and heat map analysis [23] on the software QIIME 2 [24]. Venn diagrams showing the intersection of different communities were built under R version 3.3.1.

## 3. Results

### 3.1. Determination of the Physicochemical Parameters

The soluble carbon (organic matter) which can be used as carbon source for microorganisms was below the detection limit of the instrument. The concentrations of potassium, sodium, chlorine, and soluble nitrogen, and the pH value for these samples were obtained at the same level. The concentrations of iron, magnesium, and manganese in sample Z24 collected from Anhui were generally higher than that of samples collected from Yunnan (Table 3). In contrast, the concentrations of sulfate ion and calcium in the Yunnan sample (>4000 mg/kg) were higher than those of Z24 (>1500 mg/kg) (Table 3).

The concentrations of magnesium, manganese, and sulfate ion in the sample collected from Z24 with different buried depths presented a significant difference (>2 times) (Table 3). The concentration of sulfate ion in samples XY (≈10.7 g/kg) and YYJ (≈29.0 g/kg) also presented a large difference (>2 times) (Table 3). The concentration of potassium in samples XY (311.3 g/kg) and YYJ (163.9 g/kg) was significantly different (Table 3).

### 3.2. Type of Hypersaline Environments and Microbial Communities

Salt lakes, salt mines, and solar salterns are three typical hypersaline environments found throughout the world. Among them, the salt lake, an open habitat, has a larger biomass. Due to the high fluidity of the environment, the dominant species is apparent and the nutrients are well-distributed. As compared with the salt lake, the salt mine, a closed habitat, has a smaller biomass. The salt mine is heterogenous because it has solid and buried components, and this heterogeneity favors speciation. The fluidity and habitat heterogeneity of the solar salterns are between that of the salt lake and salt mine. Additionally, the solar saltern is an open habitat, which is easily influenced by airborne and seawater microorganisms.

**Figure 2:**
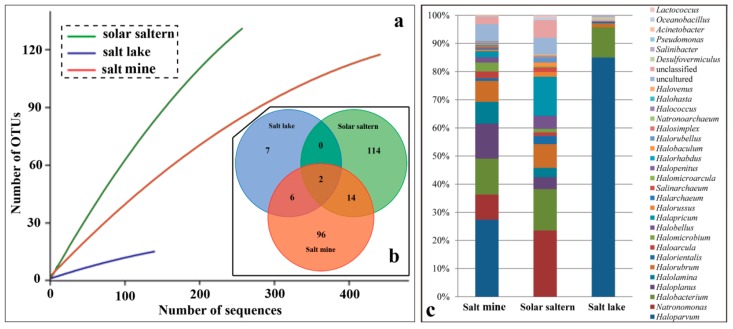
Microbial community varies from different types of hypersaline environments. (**a**) Rarefaction curve generated for 16S rRNA genes in bacterial and archaeal clones’ libraries with clusterization stringency at 97% from the salt lake (blue line), solar saltern (green line), and salt mine (red line). (**b**) Venn diagram showing the shared and unique operational taxonomic units (OTUs) (97% similarity cut off) between salt lake (blue), solar saltern (green), and salt mine (red). Analysis and diagram generation were performed using the MOTHUR v 1.3 suite of programs (http://www.mothur.org/). (**c**) Relative abundance of bacterial and archaeal community obtained from 16S rRNA gene clones’ libraries at genus level.

Microbial communities of these three types of hypersaline environments were investigated by employing clone library technology. A total of 416 clones from salt mines, 238 clones from solar salterns, and 140 clones from the salt lake were involved in operational taxonomic units (OTUs) exploration under a cut-off of 97% sequence similarity (Figure 2a). Although the rarefaction curve did not present a platform, the tendency clearly showed that OTUs from solar salterns (130) were the highest, while those of the salt lake were the lowest (15) (Figure 2a,b). OTUs from salt mine (118) were intermediate (Figure 2b). Only two OTUs were shared among these three different habitats (Figure 2b). Two OTUs were shared between the salt lake and salt mine, accounting for 13.3% and 1.7% of salt lake and salt mine’s total OTUs (Figure 2b). Eight OTUs were shared between the salt lake and solar salterns, accounting for 53.3% and 6.2% of salt lake and solar saltern’s total OTUs (Figure 2b). Sixteen OTUs were shared between the salt mine and solar salterns, accounting for 13.6% and 12.3% of the salt mine and solar saltern’s total OTUs (Figure 2b). There were abundant unique OTUs distributed in the salt mine and solar salterns (Figure 2b).

Good’s coverage for the sample of the salt lake was 0.92 which is higher than the other two habitats (salt mines and solar salterns) indicating most of the species in this sample are recognized (Table 4). The Chao1 richness estimator and the ACE richness estimator for solar salterns were 382.16 and 1085.00 respectively; the highest out of these three types of hypersaline environments (Table 4). The Simpson index for the solar saltern was 0.02, compared to a value of 0.62 for the salt lake. The Shannon index for the solar saltern was 4.36, compared to a value of 0.98 for the salt lake. As for the salt mine, both the Simpson and Shannon index values were intermediate, closer to that of solar salterns (Table 4). Based upon the species diversity values, it was obvious that the solar saltern was rich in species.

When the OTUs were assigned to the genus level taxa under the threshold of 97% sequence similarity of the 16S rRNA gene (>1.4 kb), 22 genera were uncovered from the salt mine sample, compared to 18 genera present in the solar saltern (Figure 2c). There were only seven genera uncovered from the salt lake sample (Figure 2c). The dominant genus for the salt mine and the salt lake was the same, the genus *Haloparvum*, accounting for 27.41% and 85.01% of the sequences from the salt mine and salt lake, respectively. The dominant genus in solar salterns was the genus *Natronomonas*, accounting for 23.53% of sequences from this hypersaline environment (Figure 2c). The two shared OTUs found in all three habitats were assigned to genera *Halobacterium* and *Halorubrum*, indicating that species in these two genera can survive in various hypersaline environments.

### 3.3. Geographical Isolation and Microbial Communities

**Figure 3:**
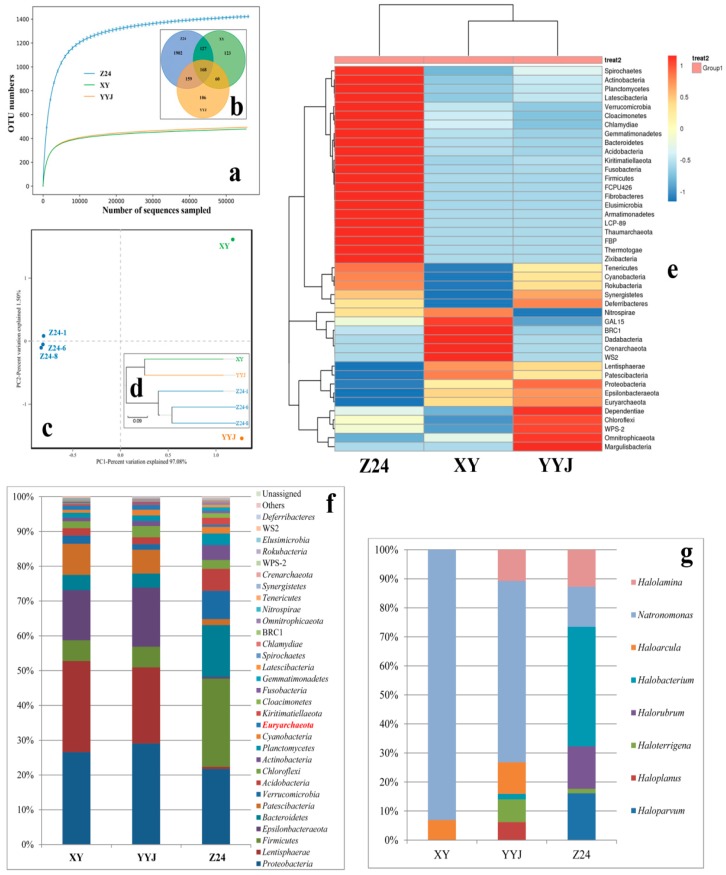
Microbial community varies from different types of salt mines. (**a**) Rarefaction curve generated from the 16S rRNA genes pyro-sequencing (97% similarity cut off). (**b**) Venn diagram showing the shared and unique OTUs (97% similarity cut off). (**c**) Principal components analysis (PCA) of three salt mines with a different location. (**d**) Clustering analysis with un-weighted pair-group method with arithmetic means (UPGMA). (**e**) Heatmap analysis showing relative abundance of microbial community of three different salt mines, as determined by high-throughput sequencing. The color code indicates the range of relative abundance for a given phylum. (**f**) Relative abundance of microbial community at the phylum level obtained from the 16S rRNA gene pyrosequencing in three different salt mines. Legends with major relative abundances (>0.1%) are shown in the figure. (**g**) Relative abundance of haloarchaeal genera in the phylum Euryarchaeota.

Samples of Z24, XY, and YYJ were all from salt mines. Samples of XY and YYJ were collected from Yunnan province (China), while sample Z24 was collected from Anhui province (China) (Figure 1). The direct distance between samples XY and YYJ was about 270 miles, while the direct distances between samples Z24 and XY and between samples Z24 and YYJ were about 1260 and 1080 miles, respectively (Figure 1). High-throughput sequencing of the V3-V4 region of the 16S rRNA gene sequences was performed for further analysis Rarefaction curves for all three salt mine samples with different geographical locations reached a perfect plateau when OTUs were defined at 97% sequence identity (Figure 3a). The total count of OTUs uncovered from samples XY, YYJ, and Z24 were 478, 493, and 2356, respectively (Figure 3b). The number of OTUs shared between samples XY and YYJ was 228 accounting for 47.7% of XY’s total OTUs and 46.2% of YYJ’s total OTUs (Figure 3b). The number of OTUs shared between samples XY and Z24 was 295 accounting for 61.7% of XY’s total OTUs and 12.5% of Z24’s total OTUs (Figure 3b). The number of OTUs shared between samples YYJ and Z24 was 327 accounting for 66.3% of YYJ’s total OTUs and 13.9% of Z24’s total OTUs (Figure 3b). There were 168 OTUs shared by all three samples (Figure 3b). The total count of OTUs for samples from XY (478) and YYJ (493) was similar and the shared OTUs (228) accounted for a relatively high percentage of the total count.

Principal component analysis (PCA) showed that samples from a single site, Z24 (Anhui, China), with different buried depths, Z24-1, Z24-6, and Z24-8, were similar in microbial community composition and clustered together which were divergent with samples of Yunnan (China), XY and YYJ (Figure 3c). UPGMA (un-weighted pair-group method with arithmetic means) and heatmap analysis also showed that samples of Yunnan, XY and YYJ were closely related and different from Z24 (Anhui, China) (Figure 3d,e). The relationship between two different phyla and samples was indicated (Figure 3e). The relative abundance of different taxa at the phylum level clearly showed that phylum Euryarchaeota (in red) in sample Z24 constituted a much greater portion of total diversity compared to that of XY or YYJ (Figure 3f). The relative abundance of different taxa at the genus level within phylum Euryarchaeota showed that few taxa at genus level were uncovered, and the genus *Natronomonas* was the only genus shared by three samples (Figure 3g). The percentage of the OTUs assigned to the genus *Natronomonas* in was highest in XY, while that of Z24 was lowest (Figure 3g). YYJ was the only site in which OTUs belonging to the genus *Haloplanus* was found, while Z24 was the only site in which OTUs belonging to genera *Haloparvum* and *Halorubrum* were found (Figure 3g).

The Chao1 richness estimator and the ACE richness estimator for sample Z24 was 2364.64 and 2366.11, respectively: higher than samples from YYJ and XY (Table 5). The Chao1 richness estimator and the ACE richness estimator for samples YYJ and XY were similar (Table 5). The Simpson index for sample Z24 was 0.01, compared to 0.05 for sample XY and 0.06 for sample YYJ. The Shannon index for sample Z24 was 6.65, compared to a value of 4.08 for both samples XY and YYJ. The indices of Simpson and Shannon illustrated that sample Z24 was richer in species (Table 5).

### 3.4. The Buried Depth and Microbial Communities

**Figure 4:**
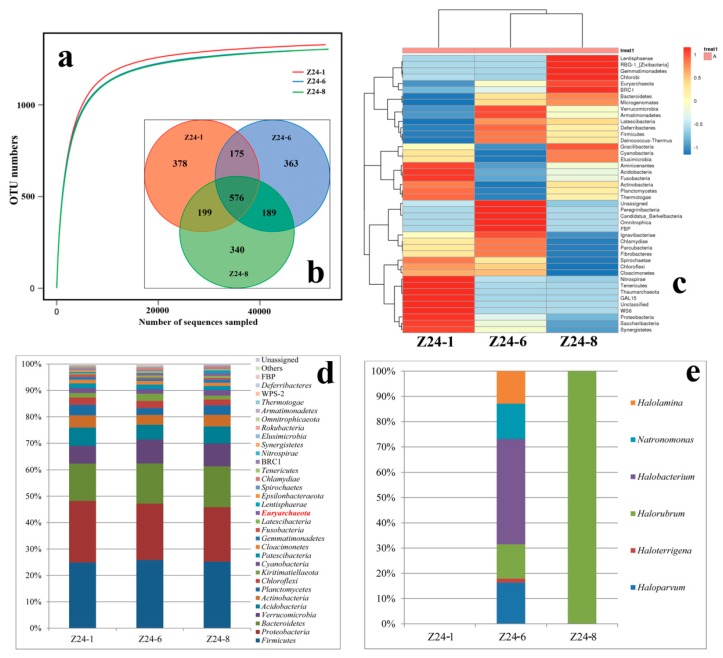
Microbial community varies from buried depths of a salt mine sample. (**a**) Rarefaction curve generated from the 16S rRNA genes pyro-sequencing (97% similarity cut off). (**b**) Venn diagram showing the shared and unique OTUs (97% similarity cut off). (**c**) Heatmap analysis showing relative abundance of microbial community of three different salt mines determined by high-throughput sequencing. The color code indicates the range of relative abundance for a given phylum. (**d**) Relative abundance of microbial community at the phylum level obtained from the 16S rRNA gene pyrosequencing in three different salt mines. Legends with major relative abundances (>0.1%) are shown in the figure. (**e**) Relative abundance of haloarchaeal genera in the phylum Euryarchaeota.

The buried depths of samples Z24-1, Z24-6, and Z24-8 were 410, 370, and 310 m underground, respectively. Rarefaction curves for all samples of Z24 with different buried depths reached a perfect plateau when OTUs were defined at 97% sequence identity (Figure 4a). Numbers of OTUs uncovered from samples Z24-1, Z24-6, and Z24-8 were very similar: there were 1328, 1303, and 1304 OTUs, respectively (Figure 4b). The number of OTUs shared between all three samples was 576, accounting for 43.4% of Z24-1’s total OTUs, 44.2% of Z24-6’s total OTUs, and 44.2% of Z24-8’s total OTUs (Figure 4b). The number of OTUs shared between samples Z24-1 and Z24-6 was 751, accounting for 56.6% of Z24-1’s total OTUs and 57.6% of Z24-6’s total OTUs (Figure 4b). The number of OTUs shared between samples Z24-1 and Z24-8 was 775, accounting for 58.4% of Z24-1’s total OTUs and 59.5% of Z24-8’s total OTUs (Figure 4b). The number of OTUs shared between samples Z24-6 and Z24-8 was 765, accounting for 58.7% of Z24-6’s or Z24-8’s total OTUs (Figure 4b). The total count of OTUs for Z24-6 (1304) and Z24-8 (1303) was almost the same. There were similar numbers of shared and unique OTUs between any pair of individual samples in the set.

The relative abundance of various phyla of sample Z24 at different depths showed that the composition and relative proportions of various phyla were very similar (Figure 4d). In Z24-1, the lowest strata, the phylum Euryarchaeota made up the smallest proportion of total OTUs (Figure 4d). Species from the class Halobacteria are found across all hypersaline environments investigated. Although the salt mine is a typical hypersaline environment, no species from the class Halobacteria were uncovered in samples from Z24-1 (collected at a buried depth of 410 m) (Figure 4e). Among these three different buried depths of sample Z24, the most haloarchaeal species were recovered from Z24-6, at an intermediate buried depth of 370 m underground (Figure 4e). Samples from Z24-8 collected from 310 m underground, closest to the surface of all salty strata, was markedly dominated by species of the genus *Halorubrum* (Figure 4e).

The Chao1 richness estimator, ACE richness estimator, Simpson index, and Shannon index for samples of Z24 from different buried depths were very close (Table 6). In general, the detailed Shannon index showed that the species diversity of the middle layer Z24-6 was richer than others (Table 6).

## 4. Discussion

### 4.1. Species Diversity Varies from Different Types of Hypersaline Environment

Saline environments, such as salt lakes, solar salterns, and salt mines, are globally distributed [25]. Halophiles thrive in hypersaline niches that are populated by both prokaryotes and eukaryotes [1]. The primer pair used for the construction of the clone library in this research was designed to be biased towards successfully amplifying the 16S rRNA gene of Archaea. However, some sequences belonging to bacterial genera, such as *Lactococcus*, *Oceanobacillus*, *Acinetobacter*, *Pseudomonas*, *Salinibacter*, and *Desulfovermiculus* were uncovered (Figure 2). Species diversity is significantly lower in the salt lake, as compared to the other two types of hypersaline environments, whether looking at data related to OTUs or genera (Figure 2). Species diversity indexes listed in Table 4 also suggest the same conclusion (Table 4). The fluidity in the salt lake is higher than others which may contribute to a more even composition and concentration of nutrients and physicochemical traits. Gene flow between two different species is more frequent in salt lakes which have higher fluidity. Compared to the salt lake, solid hypersaline environments (salt mines and solar salterns) possess a greater habitat heterogeneity which greatly promotes the process of speciation [26].

The dominant group in salt lake is much more obvious. Sequences belonging to the genus *Haloparvum* accounted for about 85% of the total sequenced 16S rRNA for the sample from Chaka salt lake, while species from this genus are completely absent in the solar saltern samples (Figure 2). 

### 4.2. Geographical Isolation Influences the Microbial Community

Clone library technology has been widely adopted to investigate the microbial community of salt mine samples [10]. Additionally, high-throughput sequencing has been widely used in recent years to investigate the prokaryotic community of different hypersaline environments [12,17]. Out of the salt mines investigated, the Dinyuan salt mine was most diverse: the total count of unique OTUs uncovered from Dinyuan was greater than the number of OTUs from XY and YYJ put together (Figure 3). The diversity indexes also showed that the species diversity in Dinyuan salt mine is greater than that of XY and YYJ, two salt mine samples collected from Yunnan, China (Table 5).

The prokaryotic communities in XY and YYJ are more similar to each other than communities in Z24 and XY or Z24 and YYJ (Figure 3e,f). Thus, in the diagram of PCA and UPGMA, niches with similar microbial communities cluster together (Figure 3c,d). The distance between XY and YYJ (≈250 km) is far less than that between ZC24 and XY or YYJ (>1500 km) (Figure 1). Geographical isolation, especially between salt mine samples from Anhui (Z24) and Yunnan (XY and YYJ), is significant. In addition, the differences in Fe, Mg, Ca, and Mn concentration in various hypersaline environments may also attribute to the variation of the microbial community (Table 3). The concentrations of elements Fe, Mg, and Mn are similar between XY and YYJ, and much lower than that of Z24. The concentration of element Ca is also similar between XY and YYJ, and is much higher than the Ca concentration of Z24 (Table 3). We speculated that the type and concentration of ions may largely modulate the pattern of the microbial community along with geographical isolation of hypersaline environments [12].

The fact that members from the phyla *Proteobacteria* were found to constitute a large proportion of different salt mines in this study agrees with previously research conducted by Xiao et al. [10] in Yunnan salt mines. *Lentisphaerae*, another dominant group in XY and YYJ, that was scarce in Z24, was not found in Xiao et al. [10]. To the best of our knowledge, this group has not yet been widely reported in hypersaline environments.

Halophilic archaea always thrive in natural salt lakes, solar salterns, and other hypersaline environments [27]. However, the extremely halophilic archaea uncovered by the high-throughput DNA sequencing approach constitute only <2% of the total OTUs in these salt mine samples. According to our findings, at the genus level, members from genus *Natronomonas* may be widely distributed throughout salt mines. In order to investigate the community of haloarchaea, haloarchaea-specific primers should be utilized instead of the universal primers for bacteria and archaea.

### 4.3. Species Diversity Along with Different Buried Depths

Different stratums of salt mines from different buried depths varied in their physicochemical traits. Obviously, the deeper stratums were formed earlier than the upper stratums. The microbial community varied from different stratums with different geological history and environmental parameters, which was similar to the pattern that the microbial community shifted along with the salinity [11,12].

The unique and shared OTUs among samples at different buried depths are very similar (Figure 4b). The diversity indexes of the samples are also similar (Table 6). Moreover, most of the phyla are generally similar (Figure 4d), suggesting that they are alike in microbial community structure with almost the same level of species diversity. However, there are still some minor differences in phylum abundance (Figure 4c) which may be attributed to different environmental parameters, such as ions and soluble nitrogen concentration, between samples from different buried depths (Table 3).

The top three dominant groups are the phyla *Firmicutes*, *Proteobacteria*, and *Bacteroides* respectively, and each of them accounted for more than 10% of the total sequences obtained (Figure 4d). According to our findings, amounts of extremely halophilic archaea in the Z24 sample series with different buried depths are not as abundant as previously recorded. Not many sequences belonging to different genera of the class *Halobacteria* have been found in this study (Figure 4e). At the bottom of the salt mine stratum (Z24-1), almost no sequences belonging to halophilic archaea were uncovered. In the middle stratum (Z24-6), sequences belonging to genera *Halolamina*, *Natronomonas*, *Halobacterium*, *Halorubrum*, *Haloterrigena*, and *Haloparvum* started to appear. In the newest stratum, only sequences affiliated to genus *Halorubrum* are present. These species are flourishing, suggesting that species of the genus *Halorubrum* may gradually outcompete other halophilic archaeal members in hypersaline environments throughout an extended period of geological time.

## 5. Conclusions

Salt lakes, solar salterns, and salt mines are three typical hypersaline environments distributed all over the planet [1]. Using clone library technology, it was discovered that species diversity in the salt lake is the lowest of these three typical environments. High-throughput sequencing reveals that geographical isolation and buried depth accompanied by the variation of the environmental parameters may largely influence the species diversity and microbial community structure. Generally, members from the phyla *Proteobacteria*, *Firmicutes*, and *Bacteroidetes* are consistently found as the dominant groups within hypersaline environments. Species belonging to the genus *Natronomonas* are widely distributed in hypersaline environments.

Future investigations of different habitats of hypersaline environments regarding fluidity, geographical isolation, and buried depth will extend our knowledge on the ecology and community structure of the bacteria and extremely halophilic archaea in these extreme environments. However, based on these results, a combination of clone library and high-throughput sequencing approaches is useful in describing the microbial community. This work provided a possibility for uncovering the living microbes along with different stratums reflecting the change of paleoclimate.

## Figures and Tables

**Table 1:** Geographic information of sampling sites.

Site	Sample	Habitat	Location	Altitude (m)	Buried Depth (m)
S1	CK	Salt lake	N 36°39′37.53″ E 99°08′49.95″	3059	0
S2	YYJ	Salt mine	N 25°16′49.61″ E 101°54′02.75″	2059	unknown
S3	XY	Salt mine	N 23°24′30.54″ E 100°39′56.51″	967	unknown
S4	HND	Solar saltern	N 19°09′23.06″ E 108°40′45.37″	35	0
S5	Z24-1	Salt mine	N 32°30′23.88″ E 117°29′44.16″	73	410
S5	Z24-6	Salt mine	N 32°30′23.88″ E 117°29′44.16″	73	370
S5	Z24-8	Salt mine	N 32°30′23.88″ E 117°29′44.16″	73	310
S6	LYG	Solar saltern	N 34°40′43.31″ E 119°13′47.07″	30	0

**Table 2:** Primers used in this study.

Name	Sequence (5′–3′)
F8	TTGATCCTGCCGGAGGCCATTG
R1462	ATCCAGCCGCAGATTCCCCTAC
F338	ACTCCTACGGGAGGCAGCA
R806	GGACTACHVGGGTWTCTAAT

**Table 3:** Physicochemical properties of salt mines.

Item	Z24-1	Z24-6	Z24-8	YYJ	XY
Fe (mg/kg)	151.7	180.8	278.9	32.79	32.17
Mg (mg/kg)	243.9	92.16	609.0	24.53	28.76
Ca (mg/kg)	1417	1259	1259	6775	4460
K (mg/kg)	198.0	304.2	332.1	163.9	311.3
Na (%)	73.32	75.07	70.22	76.40	74.13
Mn (μg/kg)	2874.24	2531.11	6970.51	775.70	891.31
Cl^−^ (mg/kg)	733,610	716,109	730,420	719,048	709,146
SO_4_^2−^ (mg/kg)	483.84	7075.31	1893.51	28,982.68	10,735.84
Soluble component (*w*/*w*)	72.13	72.13	72.13	17.8	27.2
pH	7.02	7.02	7.02	7.08	6.81
C (%)	<0.1	<0.1	<0.1	<0.1	<0.1
N (%)	0.23	0.21	0.32	0.36	0.40

**Table 4:** Species diversity indexes of three types of hypersaline environments.

Source	ACE	Chao1	Simpson	Shannon	Good’s Coverage
Salt mine	427.82	301.88	0.08	3.57	0.83
Salt lake	111.54	42.50	0.62	0.98	0.92
Solar saltern	1085.00	382.16	0.02	4.36	0.62

**Table 5:** Species diversity indexes for different salt mines.

Sample	ACE	Chao1	Simpson	Shannon
XY	511.75	534.89	0.05	4.08
YYJ	527.81	569.56	0.06	4.08
Z24	2364.64	2366.11	0.01	6.65

**Table 6:** Species diversity indexes of samples from Dinyuang salt mine under different buried depths.

Sample	ACE	Chao1	Simpson	Shannon
Z24-1	1347.94	1357.32	0.0090	6.14
Z24-6	1322.35	1322.60	0.0088	6.15
Z24-8	1332.73	1340.65	0.0089	6.09
